# A Red-Emission Fluorescent Probe with Large Stokes Shift for Detection of Viscosity in Living Cells and Tumor-Bearing Mice

**DOI:** 10.3390/molecules29091993

**Published:** 2024-04-26

**Authors:** Beilei Wang, Dezhi Yang, Xiaohong Zhong, Yuhui Liu, Yong Huang

**Affiliations:** 1 School of Pharmaceutical Engineering, Chongqing Chemical Industry Vocational College, Chongqing 401220, China; wblei1110@163.com; 2 State Key Laboratory for the Chemistry and Molecular Engineering of Medicinal Resources, School of Chemistry and Pharmaceutical Science, Guangxi Normal University, Guilin 541004, China; zxh08140202@163.com (X.Z.); liuyh9611@163.com (Y.L.); 3 School of Pharmacy, Zunyi Medical University, Zunyi 563000, China

**Keywords:** viscosity detection, large stokes shift, fluorescent imaging, cancer diagnosis

## Abstract

Abnormal viscosity is closely related to the occurrence of many diseases, such as cancer. Therefore, real-time detection of changes in viscosity in living cells is of great importance. Fluorescent molecular rotors play a critical role in detecting changes in cellular viscosity. Developing red emission viscosity probes with large Stokes shifts and high sensitivity and specificity remains an urgent and important topic. Herein, a novel viscosity-sensitive fluorescent probe (TCF-VIS1) with a large stokes shift and red emission was prepared based on the 2-dicyanomethylene-3-cyano-4,5,5-trimethyl-2,5-dihydrofuran (TCF) skeleton. Due to intramolecular rotation, the probe itself does not fluorescence at low viscosity. With the increase in viscosity, the rotation of TCF-VIS1 is limited, and its fluorescence is obviously enhanced. The probe has the advantages of simple preparation, large Stokes shift, good sensitivity and selectivity, and low cytotoxicity, which make it successfully used for viscosity detection in living cells. Moreover, TCF-VIS1 showed its potential for cancer diagnosis at the cell level and in tumor-bearing mice by detecting viscosity. Therefore, the probe is expected to enrich strategies for the detection of viscosity in biological systems and offer a potential tool for cancer diagnosis.

## 1. Introduction

Viscosity is an important microenvironmental parameter that plays a crucial role in the transport and interaction of biomolecules and related life processes [1,2]. As a key indicator of the biological microenvironment, viscosity is closely related to various physiological and pathological processes [3,4]. Abnormal changes in viscosity can lead to cellular malfunction or serve as a biomarker of the occurrence of severe diseases. For example, abnormal cell viscosity is closely related to many diseases, including inflammation, Alzheimer’s disease, and cancer [5,6,7,8]. Therefore, effective real-time detection of intracellular viscosity changes is of great significance for cancer diagnosis and treatment [9]. However, it is very challenging to detect viscosity parameters in living systems because of their complexity and real-time changes. The traditional viscometer methods mainly contain a rotary viscometer, a falling ball viscometer, and a capillary viscometer [10,11,12]. These methods are mainly based on in vitro viscosity rather than at the cellular level and do not provide the required spatial and temporal resolution [11,12,13], so it is necessary to develop a new method to detect intracellular viscosity.

In recent years, fluorescent probes have become an important tool for the viscosity monitoring of biological systems [14,15]. For most viscosity probes, the mechanism of action appears to be twisted intramolecular charge transfer (TICT) [16,17,18]. These probes typically consist of two main components: a fluorophore and a rotation group. In such probes, the fluorescence emission process is effectively quenched by the rotation motion. However, the rotation moiety itself is affected by changes in the viscosity of the surrounding medium, and when the viscosity increases, the rotation is restricted, resulting in enhanced fluorescence of the probe [19,20]. Although many viscosity-sensitive fluorescent probes have been reported, these probes sometimes have some shortcomings, including a low response signal and a small Stokes shift, and most have biological background interference for imaging due to their short emission wavelength [21,22,23,24,25]. Near-infrared fluorescence emission and large Stokes shifts could reduce the background interference, reduce the crosstalk between absorption and emission, and improve fluorescence signal-to-noise ratios [26]. Therefore, a simple and efficient viscosity-responsive fluorescent probe with a large Stokes shift and red emission for monitoring viscosity to realize cancer diagnosis is highly desirable.

**Scheme 1:**
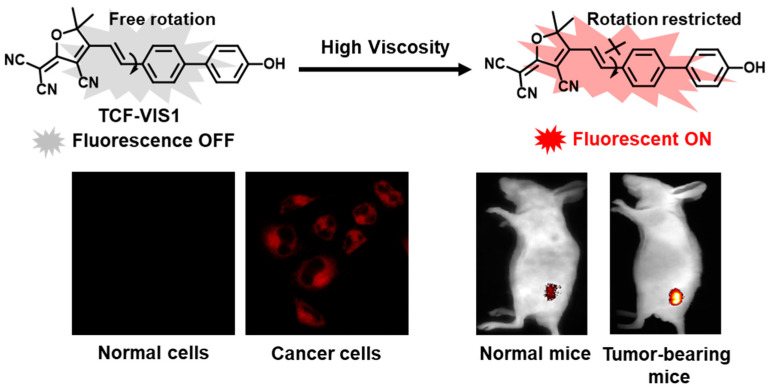
Response mechanism of probe TCF-VIS1 towards viscosity and visualization of viscosity in living cells.

To date, a large number of viscosity-sensitive fluorescent probes based on cyanine, BODIPY, naphthylamine, porphyrin, and other fluorescent dyes have been reported [5]. Moreover, 2-dicyanomethylene-3-cyano-4,5,5-trimethyl-2,5 dihydro-furan (TCF) as an excellent electron-absorbing fluorophore has attracted significant attention due to its good photostability and super-resolution imaging [27,28,29,30]. Although a variety of TCF-based fluorescent probes have been reported for biological imaging [31,32,33,34], to our knowledge, the study of TCF-based viscosity-sensitive probes is very limited [35,36,37]. In this work, a red-emission fluorescent probe, TCF-VIS1, was designed and prepared based on the TCF skeleton for viscosity detection (Scheme 1). At low viscosity, the intramolecular rotation bond of the probe can rotate, and the fluorescence is very weak. However, as the viscosity increases, the rotation process is blocked to facilitate the intramolecular charge transfer (ICT) process, so that TCF-VIS1 emits a strong NIR fluorescence signal. Thus, the viscosity can be monitored by an obvious “turn-on” fluorescence signal. In addition, TCF-VIS1 possesses a large Stokes shift (184 nm) and red emission to better eliminate background interference. It has the advantages of good sensitivity and selectivity, a large Stokes shift, and low cytotoxicity, which make it successfully used for viscosity detection in living cells and in vivo. Therefore, this work may provide a new tool for tracking viscosity-related pathological processes as well as for cancer diagnosis.

## 2. Results and Discussion

### 2.1. Syntheses of TCF-VIS1

The synthesis route of TCF-VIS1 (Scheme S1) as well as structural characterization by NMR and HRMS (Figures S11–S13) are described in Supplementary Materials.

### 2.2. Spectroscopic Response of TCF-VIS1 to Viscosity

**Figure 1:**
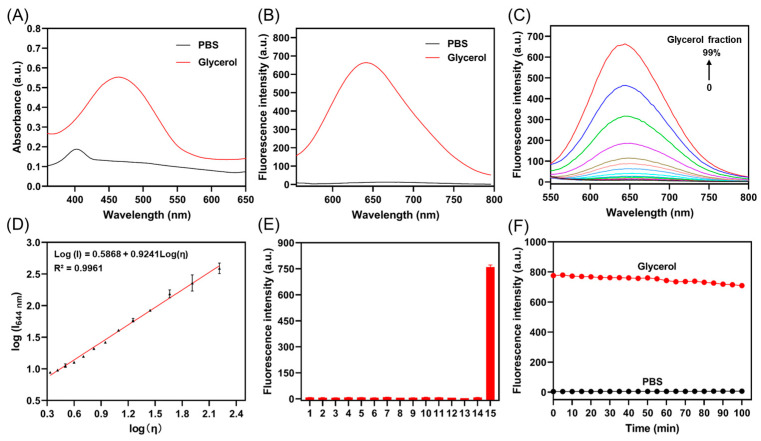
(**A**) Absorption spectra and (**B**) fluorescence spectra of TCF-VIS1 (10 μM) in PBS and glycerol. (**C**) Fluorescence spectra of TCF-VIS1 at different glycerol fractions in the glycerol–water system. (**D**) Linear relationship between log I644 nm and log η in the viscosity range of 2.15 cp–163.6 cp. (**E**) Fluorescence intensity of 10 μM TCF-VIS1 for different species (100 μM) at 644 nm: (1) PBS; (2) NO_2_^−^; (3) K^+^; (4) Mg^2+^; (5) Ca^2+^; (6) HCO_3_^−^; (7) Fe^2+^; (8) Glu; (9) Cys; (10) BSA; (11) GSH; (12) Urea; (13) H_2_S; (14) H_2_O_2_; (15) Glycerol. (**F**) The photostability of the probe TCF-VIS1 in glycerol and PBS. λ_ex_ = 460 nm, λ_em_ = 644 nm.

The photophysical properties of the probe were investigated. As shown in Figure 1A, the absorption of TCF-VIS1 in PBS was at 405 nm, and in glycerol, it was red shifted to 460 nm. As expected, TCF-VIS1 showed little fluorescence in PBS, whereas an obvious fluorescence enhancement was obtained in glycerol (Figure 1B), indicating that the probe had a good fluorescence response to viscosity. At the same time, the Stokes shift reaches 184 nm, which could effectively reduce the interference of excitation light. We then assessed the UV-Vis absorption and fluorescence properties of TCF-VIS1 in different solvents (Figure S1A,B and Table S1). The results showed that TCF-VIS1 exhibited relatively weak fluorescence emission in all solvents except glycerol, with fluorescence quantum yields ranging from 0 to 0.4. In glycerol, TCF-VIS1 displayed strong fluorescence, and the quantum yield was 0.97, indicating that TCF-VIS1 was more sensitive to viscosity than polarity.

Next, fluorescence titration of the probe TCF-VIS1 with viscosity was performed in a water–glycerol system. As shown in Figure 1C, the fluorescence intensity of TCF-VIS1 increased as the glycerol fraction (*f*_G_) gradually increased from 0 to 99%. Moreover, with the viscosity increasing from 0.89 cP to 856 cP, the fluorescence intensity of TCF-VIS1 at 644 nm is enhanced by 78 times, indicating that TCF-VIS1 is highly sensitive to viscosity. In the range of 2.15–163.6 cP, the fluorescence intensity (log I644) and viscosity (log η) of TCF-VIS1 show a good linear relationship (R^2^ = 0.9961, x = 0.9241) by fitting the Förster−Hoffmann equation (log I = 0.5868 + 0.9241 logη) (Figure 1D). Thus, the probe TCF-VIS1 can be used for the quantitative measurement of viscosity.

### 2.3. Selectivity and Photostability of TCF-VIS1 to Viscosity

To assess the selectivity of TCF-VIS1, we tested whether other constituents might potentially influence the response signals. Thirteen other species, including NaNO_2_, KCl, MgCl_2_, CaCl_2_, NaHCO_3_, FeCl_2_, Glu, Cys, BSA, GSH, Urea, H_2_S, and H_2_O_2_, were separately measured using fluorescence spectroscopy. As shown in Figure 1E, except for glycerol, TCF-VIS1 showed little response to other biologically relevant substances, indicating that TCF-VIS1 has a high viscosity selectivity and is not interfered with by other substances, which confirms the potential application of TCF-VIS1 for sensing viscosity changes in complex biological environments. In addition, we further tested the fluorescence response of TCF-VIS1 under different ions with varying concentrations (Figure S2). The results showed that, except in a high-viscosity glycerol environment, different ions did not cause remarkable changes in the fluorescence intensity of TCF-VIS1.

Next, the photostability of TCF-VIS1 under continuous laser irradiation was measured. As shown in Figure 1F, the fluorescence intensity of probe TCF-VIS1 in glycerol was stable at 644 nm with continuous irradiation for 60 min, indicating that the probe had good photostability and has the potential to be used for bioimaging applications.

### 2.4. Effect of pH on TCF-VIS1 Response Viscosity

The effect of pH on the fluorescence of TCF-VIS1 was also evaluated in PBS and 40% PBS + 60% glycerol, respectively. As shown in Figure S3, the fluorescence intensity of TCF-VIS1 hardly varied in the pH range of 4.0~10.0 in both low-viscosity and high-viscosity media, suggesting that TCF-VIS1 could be used to monitor the viscosity in a wide pH range. Thus, TCF-VIS1 could be further applied in biological systems.

### 2.5. Density Functional Theory Calculations

**Figure 2:**
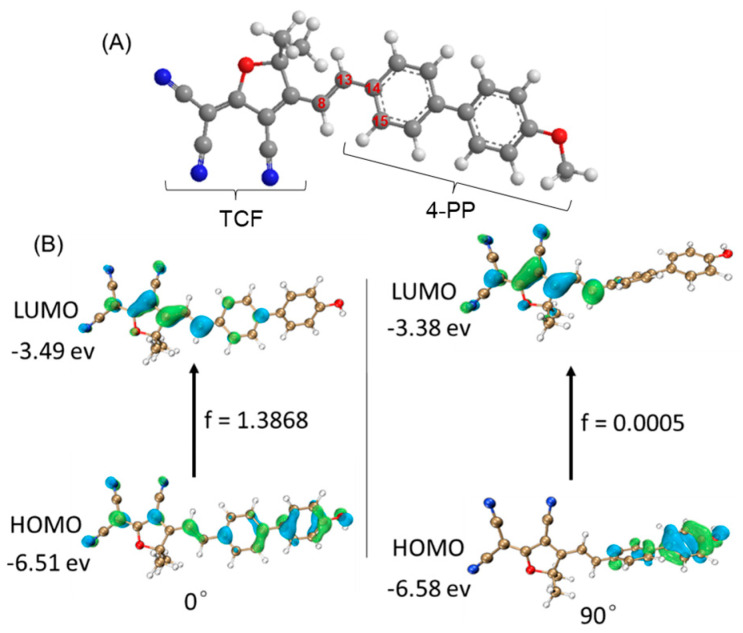
(**A**) The optimized geometry of TCF-VIS1 in an excited state. (**B**) Calculated frontier molecular orbitals of TCF-VIS1 with dihedral angles of 0° and 90° at around C8−C13−C14−C15, respectively.

To further understand the optical properties of probe TCF-VIS1, the DFT/TDDFT calculation was performed using the B3LYP/def2tzvp em = gd3bj method via Gaussian 09. As shown in Figure 2, when the dihedral angle at around C8−C13−C14−C15 was either 0° or 90°, the HOMO was centered at 4-PP, and the LUMO was centered at the TCF core. These phenomena suggest that TCF-VIS1 undergoes an intramolecular charge transfer (ICT) process when it is excited from the ground state to the excited state. Furthermore, when the dihedral angle at around C8−C13−C14−C15 was 0°, the probe TCF-VIS1 maintained a planar conformation with an oscillator strength (f) of 1.3868. However, when the dihedral angle at around C8−C13−C14−C15 was 90°, the probe kept the orthogonal conformation in a twisted intramolecular charge transfer (TICT) state, and the oscillator strength was only 0.0005. These results showed that the probe TCF-VIS1 could form a TICT state through intramolecular rotation, which had a weak fluorescence emission. Therefore, considering the rotation of the single bond in the π system of the probe TCF-VIS1, the TICT state of TCF-VIS1 would be formed by intramolecular rotation in a low-viscosity environment, resulting in a decrease in fluorescence. However, when the viscosity increases, limiting the rotation of the intramolecular single bonds, the ICT effect within the molecule causes the probe to emit fluorescence. Therefore, TCF-VIS1 could be used to detect changes in viscosity.

### 2.6. Cytotoxicity and Localization

Based on the above results, we further evaluated the ability of TCF-VIS1 to detect viscosity in living cells. Firstly, the cytotoxicity of the probe was evaluated in living HepG-2 cells and HeLa cells by MTT assay. As displayed in Figure S4, the survival rate of cells at a 40 μM concentration was more than 83%, indicating that the probe had low cytotoxicity. Furthermore, the photostability of TCF-VIS1 in cells was studied. The imaging results showed that there was no significant change in fluorescence imaging under continuous light irradiation (Figure S5), suggesting that TCF-VIS1 has good photostability in fluorescence imaging.

**Figure 3:**
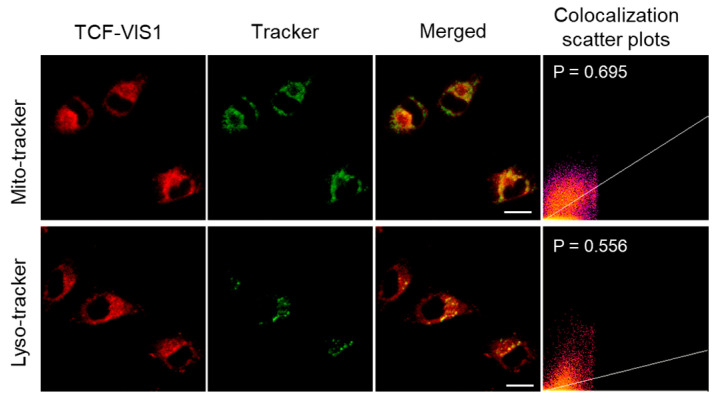
Fluorescent confocal images of HepG-2 cells with TCF-VIS1 and a tracker. TCF-VIS1: λ_ex_ = 488 nm, λ_em_ = 600~750 nm. Tracker: λ_ex_ = 488 nm, λ_em_ = 500~520 nm. Merge: the merged images of the green channel and the red channel. Colocalization scatter plots: the scatter plot of the green channel and the red channel. Scale bar: 20 μm.

Next, the distribution of TCF-VIS1 in lysosomes and mitochondria was studied using commercial Lyso-Tracker Green and Mito-Tracker Green dyes. As shown in Figure 3, the Pearson colocalization coefficients (P) of TCF-VIS1 with mitochondria and lysosomes in HepG-2 cells were 0.695 and 0.556, respectively, indicating that TCF-VIS1 has similar moderate colocalization imaging with mitochondria and lysosomes. Similar distribution results were further confirmed in HeLa cells (Figure S6). These results showed that TCF-VIS1 can be distributed in both mitochondria and lysosomes but cannot remain in specific subcellular organelles.

### 2.7. Monitoring the Cellular Viscosity

**Figure 4:**
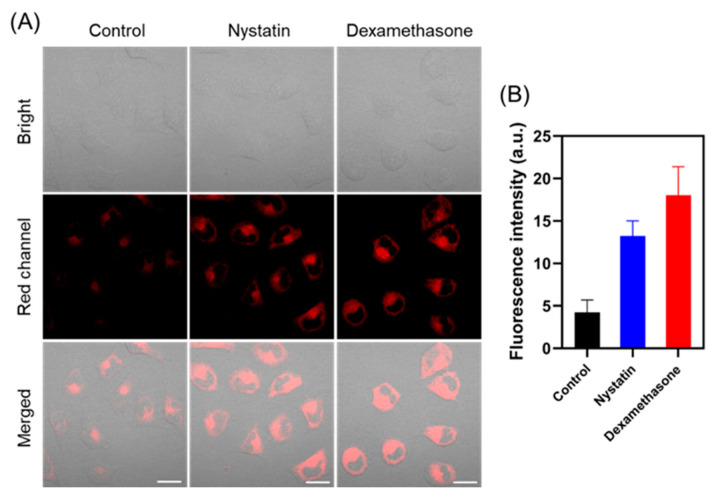
Fluorescence images of HepG-2 cells: (**A**) HepG-2 cells were incubated with TCF-VIS1 (10 μM) for 10 min as a control; HepG-2 cells were treated with nystatin (10 μM) or dexamethasone (10 μM) for 45 min and then incubated with TCF-VIS1 (10 μM) for another 10 min. λ_ex_ = 488 nm, λ_em_ = 600~750 nm. Scale bar: 20 μm. (**B**) Fluorescence intensities in panel (**A**), which were obtained by Image J (Fiji).

Subsequently, TCF-VIS1 was used to detect cellular viscosity changes. Dexamethasone and nystatin are well known to enhance the viscosity of lysosomes and mitochondria [10]. Since TCF-VIS1 can be distributed in lysosomes and mitochondria, dexamethasone and nystatin were used as stimulators of cellular viscosity. As displayed in Figure 4, HepG-2 cells pretreated with nystatin and dexamethasone, respectively, before TCF-VIS1 staining showed significantly enhanced fluorescence in the red channel compared with the control cells only incubated with TCF-VIS1. Similarly, TCF-VIS1 was also able to monitor viscosity changes in HeLa cells after nystatin or dexamethasone treatment (Figure S7). These results suggest that TCF-VIS1 can be used to monitor changes in intracellular viscosity.

Besides, HepG-2 cells were incubated with nystatin at different concentrations (0, 10, 40, and 80 μM) for 30 min and then incubated with TCF-VIS1. As shown in Figure S8, the fluorescence of the red channel was enhanced gradually with the increase in nystatin concentration. In addition, as lipopolysaccharide (LPS) is a classic inflammatory inducer that can induce cellular inflammation and lead to increased cellular viscosity [38], we selected LPS as a stimulus to further verify the ability of TCF-VIS1 to monitor viscosity changes in cells. HepG-2 cells were preincubated with different concentrations of LPS and then treated with TCF-VIS1. As can be seen from Figure S9, the fluorescence of the red channel was gradually enhanced with the increase in LPS concentration. These results further suggest that TCF-VIS1 is an effective tool for detecting cellular viscosity changes.

### 2.8. Monitoring the Viscosity during Ferroptosis

**Figure 5:**
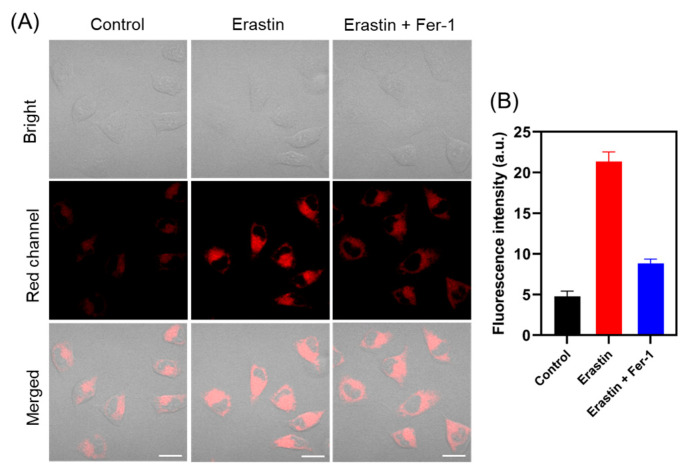
(**A**) Fluorescence images of HepG-2 cells treated with TCF-VIS1 (10 μM). Control: cells treated with TCF-VIS1; Erastin: cells treated with erastin (10 μM), followed by TCF-VIS1; Erastin + Fer-1: cells pretreated with Fer-1 (10 μM) and Erastin (10 μM), followed by TCF-VIS1. (**B**) Relative fluorescence intensity of different cells in panel (**A**). Scale bar: 20 µm. (λ_ex_ = 488 nm; λ_em_ = 600~750 nm).

We further investigated the viscosity changes during ferroptosis, as studies have shown that during ferroptosis, the conversion of unsaturated lipids to lipid peroxides increases the cell viscosity [39,40]. As displayed in Figure 5, HepG-2 cells pretreated with erastin (a ferroptosis activator) showed an obvious 4.5-fold fluorescence enhancement in the red channel, indicating a significant increase in viscosity during ferroptosis. In addition, when HepG-2 cells were simultaneously pretreated with erastin and Fer-1 (a ferroptosis inhibitor), the fluorescence in the red channel showed little change compared with the control group, indicating that the change in viscosity was correspondingly reduced during the inhibition of ferroptosis. Therefore, the probe is expected to be used to monitor the physiological and pathological processes related to viscosity.

### 2.9. Differentiation of Normal Cells and Tumor Cells

**Figure 6:**
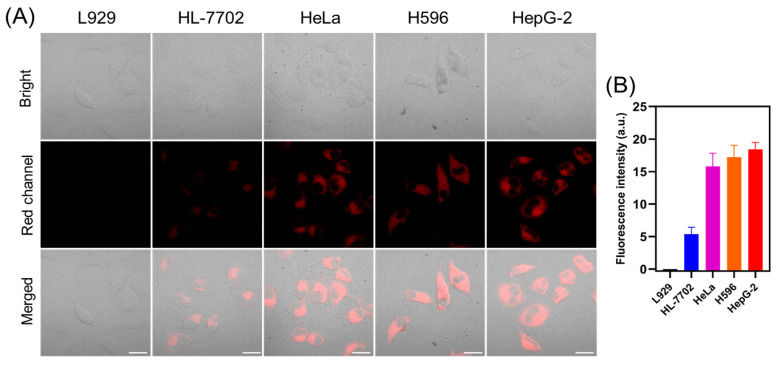
(**A**) TCF-VIS1 (10 μM) staining for imaging of normal cells (L929 and HL-7702) and cancer cells (HeLa, H596, and HepG-2). Scale bar: 20 µm. (λ_ex_ = 488 nm; λ_em_ = 600~750 nm). (**B**) The relative fluorescence intensities of the images in panel (**A**).

Previous studies have shown that cancer cells are more viscous than normal cells [41]. Therefore, both normal cells (L929, HL-7702) and cancer cells (HeLa, H596, HepG-2) are stained with TCF-VIS1. As shown in Figure 6, the probe showed significantly enhanced red fluorescence in tumor cells and weaker fluorescence in normal cells, indicating that TCF-VIS1 can successfully distinguish tumor cells from normal cells. In addition, fluorescence imaging was also performed on L929 cells and mouse breast cancer cells (4T1), and it was found that the probe TCF-VIS1 had obvious red fluorescence in 4T1 cells, while it was difficult to observe fluorescence in L929 cells (Figure S10). Therefore, these results suggest that TCF-VIS1 has the potential for cancer diagnosis by detecting differences in intracellular viscosity.

### 2.10. Cancer Diagnosis In Vivo

**Figure 7:**
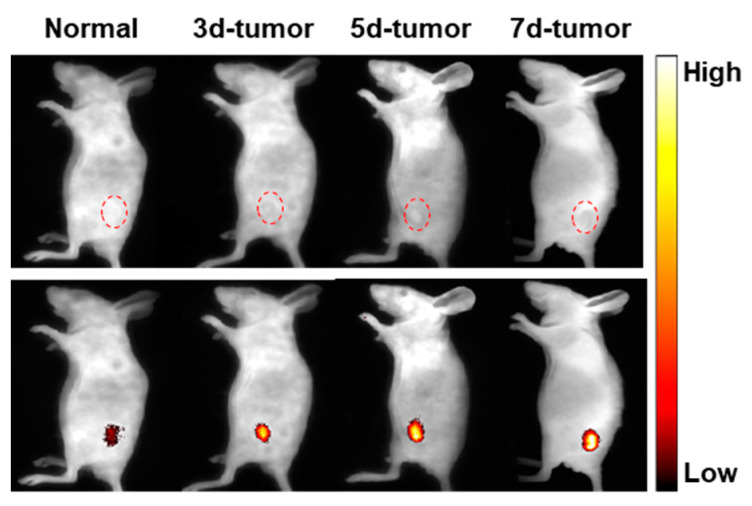
In vivo fluorescence imaging of TCF-VIS1 was performed in normal mice and in tumor-bearing mice on days 3, 5, and 7 after inoculation with 4T1 cells.

We further prepared a 4T1 tumor-bearing mouse model to study the response of TCF-VIS1 to viscosity in vivo. As displayed in Figure 7, in normal mice, the probe TCF-VIS1 exhibited only slight fluorescence. In contrast, the fluorescence at the tumor site of 4T1 tumor-bearing mice with different days was significantly enhanced, demonstrating that TCF-VIS1 could be a useful tool for visualizing tumors by monitoring viscosity in vivo.

## 3. Materials and Methods

### 3.1. Reagents and Apparatus

All solvents and reagents used were reagent-grade and were used without further purification. Nystatin, dexamethasone, and 3-(4,5-dimethyl-2-thiazolyl)-2,5-diphenyl tetrazolium bromide (MTT) were purchased from Aladdin (Shanghai, China). Lipopolysaccharides (LPS) were obtained from Macklin (Shanghai, China). Lyso-Tracker Green and Mito-Tracker Green were obtained from Beyotime Biotechnology (Shanghai, China). Dulbecco’s Modified Eagle Medium (DMEM) was purchased from Thermo Fisher (Gibco, Billings, MT, USA). Fetal bovine serum (FBS) was purchased from Gemini (New York, NY, USA). Trypsin with EDTA and penicillin/streptomycin were obtained from Solarbio (Beijing, China).

^1^H NMR and ^13^C NMR spectra were measured on an Agilent 400 MHz DDZ spectrometer. Mass spectrometric data were recorded in positive mode with a Thermo Scientific Exactive LC-MS instrument (Waltham, MA, USA). Absorption spectra were measured and recorded on an Agilent Cary 60 UV-Visible Spectrophotometer (Agilent Technologies, Santa Clara, CA, USA). Fluorescence spectra were obtained with an LS-55 fluorescence spectrophotometer (Perkin-Elmer, Waltham, MA, USA). Fluorescence imaging was carried out on a LSM710 confocal laser scanning microscope (Zeiss, Oberkochen, Germany). The MTT assay was made on an EL × 800 Microplate Reader (BioTek, Shoreline, WA, USA). Human cervical cancer cells (HeLa), human lung squamous cell carcinoma (H596), human liver cancer cells (HepG-2 cells), human normal liver cells (L02 cells), mouse fibroblast cells (L929), and mouse breast cancer cells (4T1) were supplied by the Committee on Type Culture Collection of the Chinese Academy of Sciences (Shanghai, China).

### 3.2. Synthesis of the Florescent Probe TCF-VIS1

#### 3.2.1. Synthesis of Compound **1**

To a solution of 4-bromobenzaldehyde (3.0 g, 16.2 mmol) and (4-hydroxyphenyl)boronic acid (2.7 g, 19.5 mmol) in 60 mL of THF, 2M K_2_CO_3_ (15 mL) and tetra-(triphenylphosphine)palladium (940 mg, 5% mmol) were added. The mixture was stirred at 60 °C under a nitrogen atmosphere for 8 h. After that, the reaction mixture was poured into 30 mL of water, and the resulting mixture was extracted with DCM (50 mL × 3). The combined extracts were washed with brine, dried over anhydrous Na_2_SO_4,_ and evaporated to afford crude, which was purified by column chromatography (EA/PE = 1/20~1/10) to yield compound **1**. Yield: 2.4 g (75%). ^1^H NMR (400 MHz, CDCl_3_) δ 9.92 (s, 1H), 7.91 (d, *J* = 8.2 Hz, 2H), 7.73 (d, *J* = 8.2 Hz, 2H), 7.52 (d, *J* = 8.8 Hz, 2H), 6.97 (d, *J* = 8.8 Hz, 2H), 4.95 (s, 1H).

#### 3.2.2. Synthesis of Compound **2**

In absolute ethanol (20 mL), there was dissolved Na (200.0 mg, 8.6 mmol). The mix was stirred for 0.5 h at room temperature. Then, the 3-hydroxy-3-methylbutan-2-one (5.0 g, 58.6 mmol) and malononitrile (8.0 g, 120 mmol) were added, and the mixture was stirred at room temperature for 1.5 h. In the next step, the absolute ethanol (20 mL) was added, and the mixture was refluxed for another 1 h. After the reaction was completed, the mix was cooled, filtered off, washed with cold ethanol, and dried in a vacuum to obtain compound **2**. Yield: 6.3 g (65%). ^1^H NMR (400 MHz, CDCl_3_) δ 2.46 (s, 3H), 1.79 (s, 6H).

#### 3.2.3. Synthesis of TCF-VIS1

To a solution of compound **1** (2.2 g, 11 mmol) and compound **2** (2.0 g, 10 mmol) in the THF/EtOH (4:1, 20 mL), ammonium acetate (780 mg, 10 mmol) was added. The mixture reacted at room temperature for 1.5 h. As soon as the reaction was completed, the solvent was concentrated under reduced pressure to obtain the residue, which was purified by column chromatography on silica gel with eluent dichloromethane/methanol (*v*/*v*, 100:1) to produce compound TCF-VIS1. Yield: 2.1 g (55%). ^1^H NMR (400 MHz, DMSO-d_6_) δ 9.81 (s, 1H), 7.98 (d, *J* = 8.0 Hz, 2H), 7.94 (d, *J* = 16.0 Hz, 1H), 7.77 (d, *J* = 8.0 Hz, 2H), 7.65 (d, *J* = 8.0 Hz, 2H), 7.24 (d, *J* = 16.0 Hz, 1H), 6.89 (d, *J* = 8.0 Hz, 2H), 1.81 (s, 6H). ^13^C NMR (101 MHz, DMSO-d_6_) δ 177.60, 175.67, 158.65, 147.61, 144.20, 132.85, 130.70 (2C), 129.76, 128.60 (2C), 126.81 (2C), 116.41 (2C), 115.04, 113.23, 112.39, 111.47, 99.83, 99.21, 54.63, 25.62 (2C). ESI-HRMS Calc. for C_24_H_17_N_3_O_2_, 379.1321, found [M − H]^−^: 378.1248.

### 3.3. Determination of Viscosity and Fluorescence Spectral Analysis

First, a TCF-VIS1 solution (1.0 mM) was prepared in DMSO. Viscosity determination was performed in the 1 mL incubation mixture containing the stock solution (10 µL) and solvent mixture (990 µL, water–glycerol solvent systems) to obtain the final TCF-VIS1 concentration (10.0 µM). These solutions were measured with a fluorescence spectrophotometer. λ_ex_ = 460 nm.

### 3.4. The Förster–Hoffmann Equation

The relationship between the fluorescence emission intensity of the probe and the solvent viscosity could be formulated by the Förster–Hoffmann equation as follows:

log I = C+ xlogη

where η is the viscosity, I is the emission intensity, C is a constant, and x is the sensitivity of the probe to viscosity [19].

### 3.5. Computational Methods

All molecular configurations were modeled using chemdraw, and initial structural optimization was performed using chem3D under the MM2 force field and saved in mol2 format. Thereafter, the gaussian software (Gaussian 09) is used to calculate the structure optimization and frequency at the level of B3LYP/6-31G** em = gd3bj based on density functional calculation, and then the energy calculation is carried out by B3LYP/def2tzvp em = gd3bj. Finally, based on time-dependent density functional theory (TDDFT), states were set as 20 in B3LYP/def2tzvp em = gd3bj to calculate the structure of excited states and fluorescence spectrum information. All data were processed using Multiwfn software (Multiwfn 3.7) [42] and VMD software (Version 1.93).

### 3.6. Colocalization Imaging Experiments

Cells were treated with TCF-VIS1 (10 μM) for 10 min and washed with PBS. Mito-Tracker Green and Lyso-Tracker Green were employed to stain cell mitochondria and lysosomes, respectively. Then, fluorescence images were recorded on a confocal laser scanning microscope (CLSM).

### 3.7. Fluorescence Imaging in Living Cells

HepG-2 cells and HeLa cells were cultured in DMEM and 1640 medium supplemented with 10% FBS at 37 °C under a 5% CO_2_ atmosphere, respectively. The cells were then seeded in a glass bottom dish and incubated overnight. Then, it was cultured with dexamethasone or nystatin (10 μM) for 30 min, treated with the TCF-VIS1 (10 μM) probe at 37 °C for 10 min, and washed with PBS 3 times. Fluorescence imaging was performed on the CLSM.

Cancer cells (HeLa, H596, HepG-2, 4T1) and normal cells (HL-7702 cells, and L929) were treated with TCF-VIS1 (10 μM) for 10 min, washed with PBS, and imaged on CLSM (λ_ex_ = 488 nm, λ_em_ = 600~750 nm).

### 3.8. In Vivo Fluorescence Imaging

All animal experiments were conducted in accordance with the guiding principles of the Experimental Animal Ethics Committee of Guangxi Normal University (No. 202203-003). Moreover, 1 × 10^6^ 4T1 cell suspensions were implanted subcutaneously in the left hind flank of each mouse. TCF-VIS1 (50 μM, 50 µL) was intratumorally injected into 4T1 tumor-bearing mice. Fluorescence imaging was performed using the FXPRO in vivo imaging system (λ_ex_ = 470 nm, λ_em_ = 700 nm).

## 4. Conclusions

In summary, we prepared a novel red emission viscosity-sensitive fluorescence probe, TCF-VIS1, with a large Stokes shift (184 nm) based on the TCF skeleton. The fluorescence intensity of the probe increased significantly with the increase in viscosity, and the response mechanism of TCF-VIS1 toward viscosity was verified by TDDFT calculation. The probe has the advantages of simple preparation, excellent photostability, good sensitivity and selectivity, and low cytotoxicity, which make it successfully used for sensing viscosity in living cells. The results demonstrated that there is an increase in the cellular viscosity of the cells upon treatment with nystatin and dexamethasone (stimulators of cellular viscosity), respectively. In addition, TCF-VIS1 was successfully used to indicate ferroptosis and distinguish cancers from normal cells by detecting viscosity in living cells. Moreover, TCF-VIS1 showed its potential for cancer diagnosis in tumor-bearing mice. These findings suggest that TCF-VIS1 is expected to enrich strategies for the detection of viscosity in biological systems and offer a promising tool for cancer diagnosis.

## Data Availability

Data are contained within the article.

## Supplementary Materials

The following supporting information can be downloaded at: https://www.mdpi.com/article/10.3390/molecules29091993/s1. Scheme S1: Synthesis routes of probe TCF-VIS1. Figure S1: UV–Vis and fluorescent response of TCF-VIS1 towards different solvents. Table S1: The spectroscopic property data of TCF-VIS1 in different solvents. Figure S2: Fluorescence spectra of TCF-VIS1 in the presence of different ions. Figure S3: Fluorescence spectra of TCF-VIS1 at different pHs. Figure S4: Cytotoxicity of TCF-VIS1. Figures S5–S10: Fluorescent imaging experiments. Figures S11–S13: ^1^H NMRS, ^13^C NMRS and HRMS of TCF-VIS1. Table S2: Previous viscosity probes and this work. Refs. [8,9,10,11,18,43,44,45,46,47] are cited in the Supplementary Materials.
