# Supplementary material for: A Red-Emission Fluorescent Probe with Large Stokes Shift for Detection of Viscosity in Living Cells and Tumor-Bearing Mice
Source: Molecules. 2024 Apr 26;29(9):1993. doi: 10.3390/molecules29091993 (PMC11085742; doi:10.3390/molecules29091993)
Supplement: Supplementary file 1 [file molecules-29-01993-s001.zip › molecules-2955142-supplementary.pdf]

Supporting Information

A Red-Emission Fluorescent Probe with Large Stokes Shift for Detection of Viscosity in Living Cells and Tumor-Bearing Mice

Beilei Wang [1,2], Dezhi Yang [3,*], Xiaohong Zhong [2], Yuhui Liu [2] and Yong Huang [2,*]

[1] School of Pharmaceutical Engineering, Chongqing Chemical Industry Vocational College, Chongqing 401220, China; wblei1110@163.com
[2] State Key Laboratory for the Chemistry and Molecular Engineering of Medicinal Resources, School of Chemistry and Pharmaceutical Science, Guangxi Normal University, Guilin 541004, China; zxh08140202@163.com (X.Z.); liuyh9611@163.com (Y.L.)
[3] School of Pharmacy, Zunyi Medical University, Zunyi 563000, China
* Correspondence: lpydz@163.com (D.Y.); huangyong_2009@163.com (Y.H.)

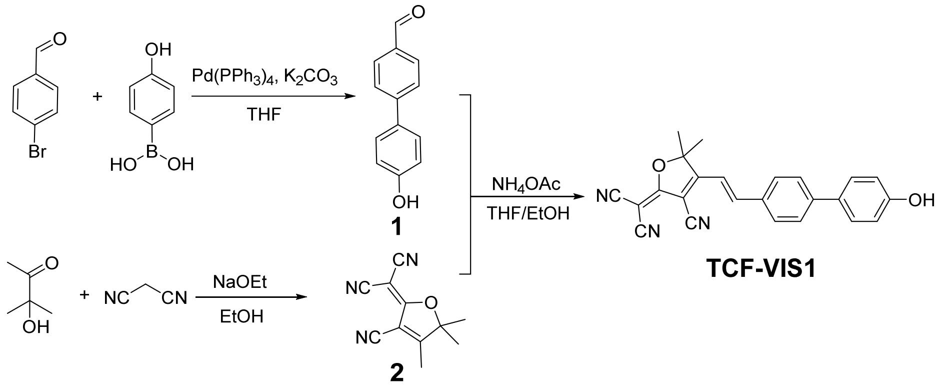

Scheme S1. Synthesis routes of probe TCF-VIS1.

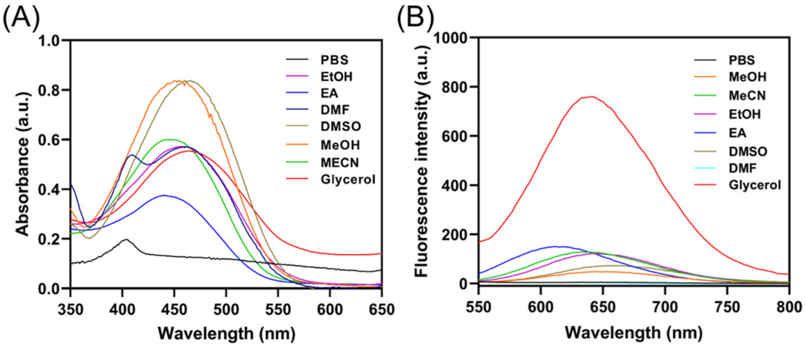

Figure S1. (A) Absorption spectra of TCF-VIS1 (10 μM) in different solvents. (B) The fluorescence spectra of TCF-VIS1 (10 μM) in different solvents (λ_{ex}= 460 nm)

Table S1. The spectroscopic properties data of probe TCF-VIS1 in different solvent.

Solvent	ξ	η^a (cp)	λ_{ab} (nm)	λ_{em} (nm)	Stokes shift (nm)	Φ^b
Glycerol	42.5	1150	460	644	184	0.9661
EtOH	24.3	1.2	450	640	190	0.2679
MeOH	33.6	0.59	455	650	195	0.01682
DMSO	48.9	2.24	465	665	200	0.0440
EA	6.02	0.45	442	612	170	0.4261
MeCN	37.5	0.37	443	638	195	0.1706
DMF	37.6	0.80	460	—[c]	—[c]	0.0230
H_2O	80.4	1.01	405	—[c]	—[c]	0.1537

[a] Viscosity of the solvent. [b] Quantum yield was reported in % and measured with steady state and transient state fluorescence spectrometer.

[c] No fluorescence was observed.

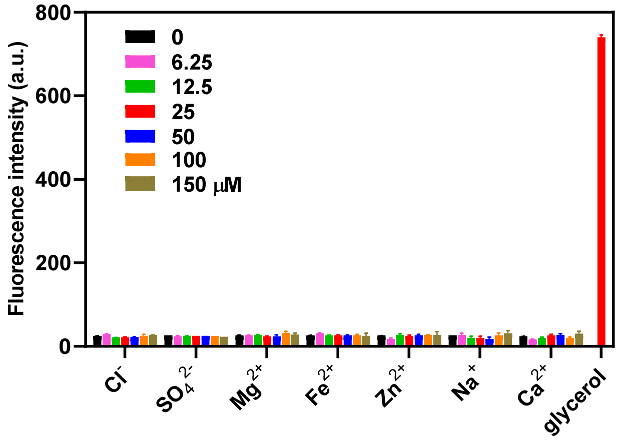

Figure S2. TCF-VIS1 (10 μM) in glycerol or PBS mixtures in the presence of different ions with varies concentrations.

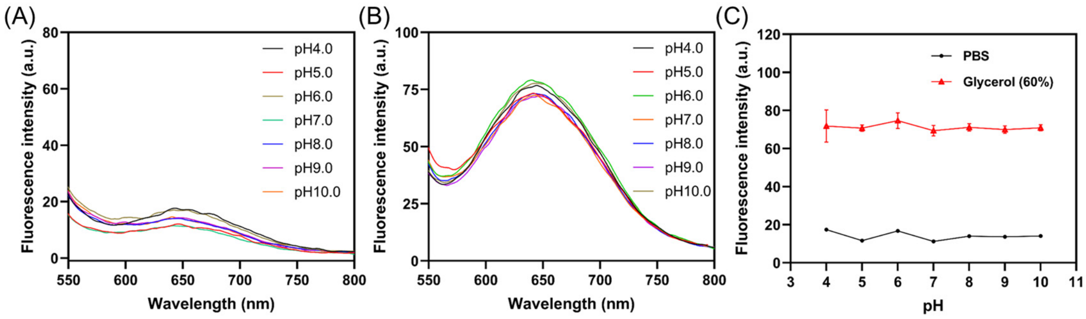

Figure S3. The fluorescence spectra of TCF-VIS1 (10 μM) in (A) PBS and (B) 60% glycerol with different pH, respectively. λ_{ex}= 460 nm. (C) The effect of pH on the fluorescence emission intensity at 644 nm of TCF-VIS1 in PBS and glycerol (f_G = 60%).

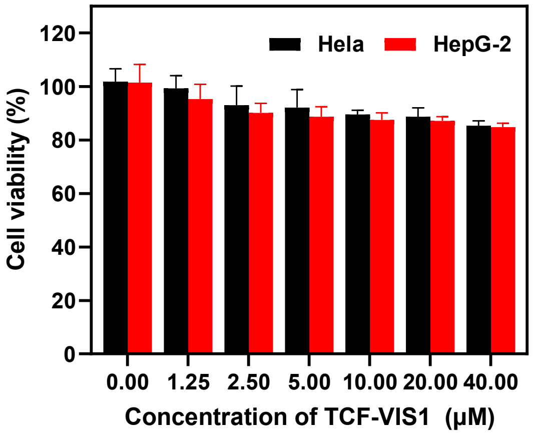

Figure S4. Cytotoxicity assays of TCF-VIS1 toward Hela cells and HepG-2 cells for 24 h incubation.

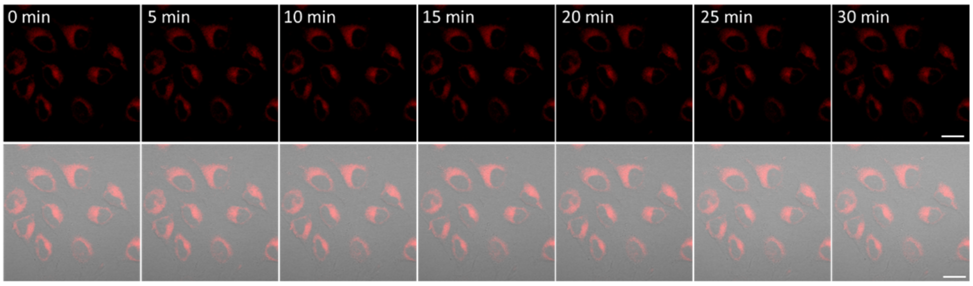

Figure S5. Photostability of TCF-VIS1 (10 μM) in Hela cells. λ_{ex} = 488 nm, λ_{em} = 600 ~ 750 nm. Scale bar: 20 μm.

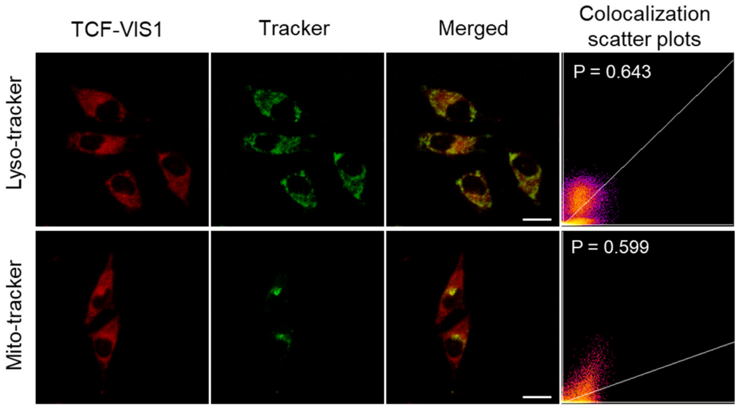

Figure S6. Fluorescent confocal images of HeLa cells with TCF-VIS1 and Tracker. Green Channel: fluorescence images of Mito-Tracker Green or Lyso-Tracker Green. Red Channel: fluorescence images of TCF-VIS1 (λ_{ex} = 488 nm; λ_{em} = 600 ~ 750 nm). Merge: the merged images of green channel and red channel. Colocalization scatter plot: the scatter plot of green channel and red channel. Scale bar: 20 μm.

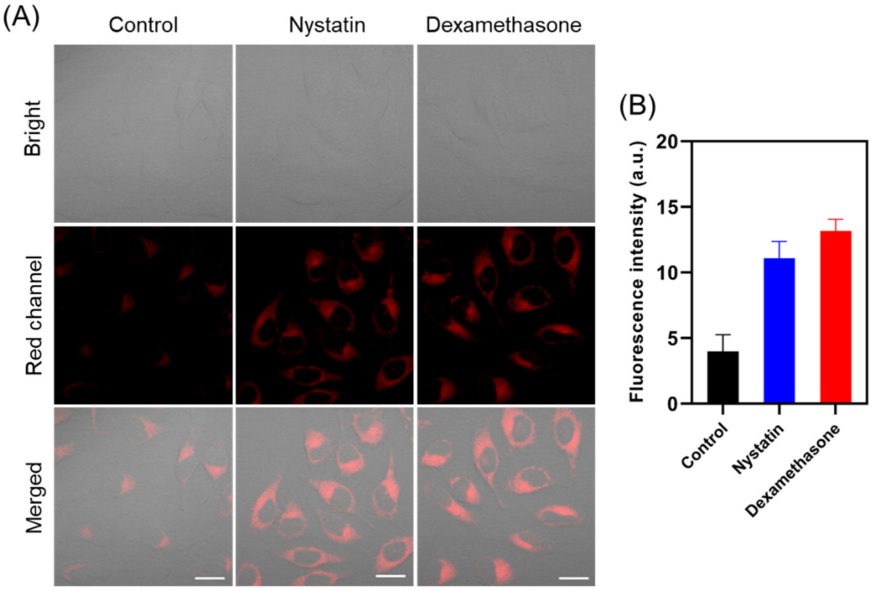

Figure S7. Confocal laser fluorescence images of HeLa cells: (A) HeLa cells were

incubated with TCF-VIS1 (10 μM) for 10 min is for control; HeLa cells were incubated with nystatin (10 μM) or dexamethasone (10 μM) for 45 min, and then treated with TCF-VIS1 (10 μM) for another 10 min. λ_{ex} = 488 nm, λ_{em} = 600 ~ 750 nm. Scale bar: 20 μm. (B) Fluorescence intensities in panel A, which were obtained by Image J.

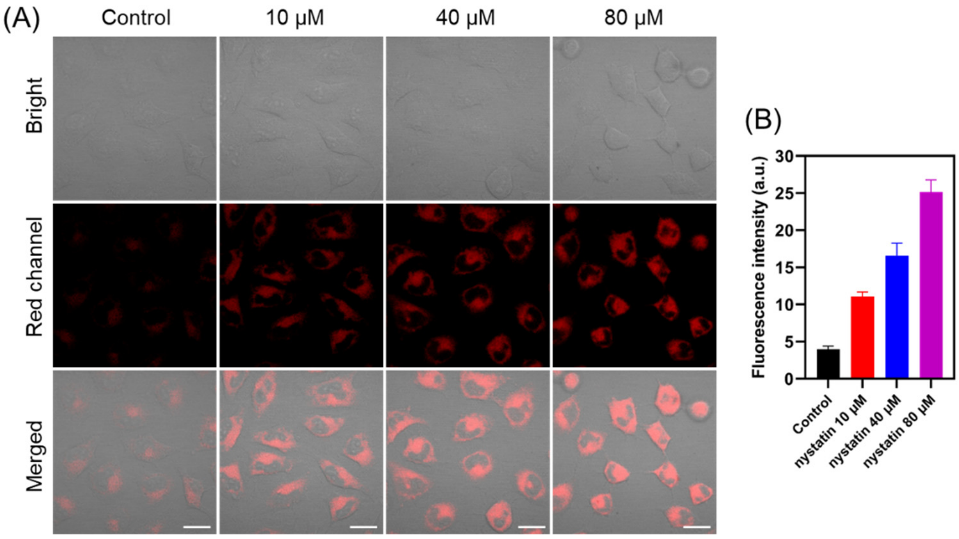

Figure S8. (A) CLSM images of living HepG-2 cells treated with different concentration of nystatin, then stained with TCF-VIS1 (10 μM) for 10 min. (B) Fluorescence intensities of images obtained from (A). λ_{ex} = 488 nm, λ_{em} = 600 ~ 750 nm. Scale bar: 20 μm.

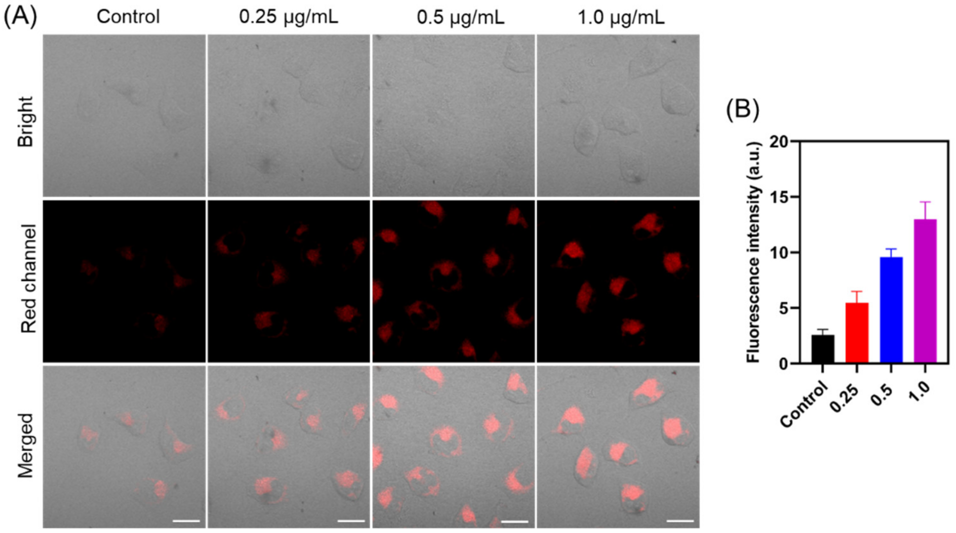

Figure S9. (A) CLSM images of living HepG-2 cells treated with different concentration of LPS, then stained with TCF-VIS1 (10 μM). (B) Fluorescence intensities of images obtained from (A). λ_{ex} = 488 nm, λ_{em} = 600 ~ 750 nm. Scale bar: 20 μm.

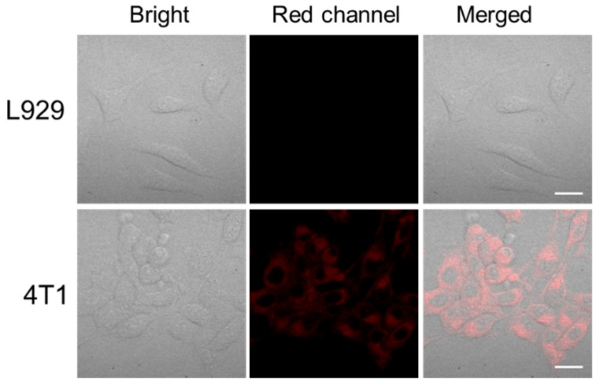

Figure S10. Fluorescence images of L929 cells and 4T1 cells treated with TCF-VIS1 (10 μM), respectively. Scale bar: 20 μm. (λ_{ex} = 488 nm; λ_{em} = 600 ~ 750 nm).

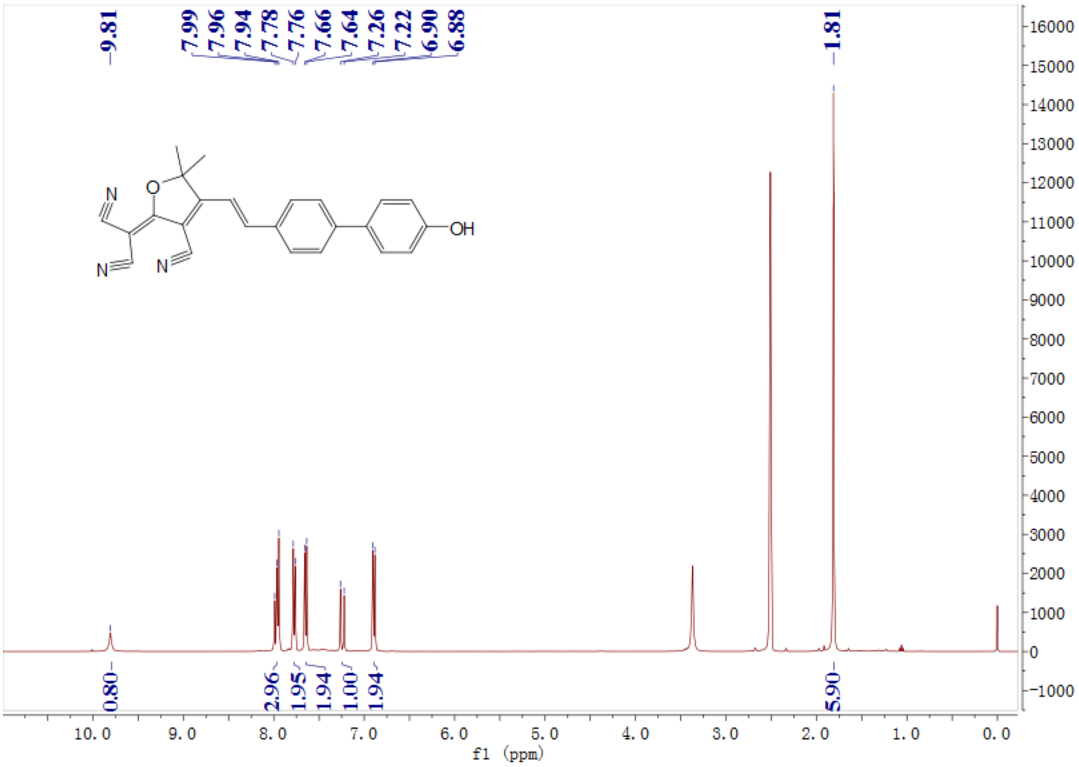

Figure S11. ^{1}H NMR (400 MHz, DMSO-d_6) spectrum of TCF-VIS1.

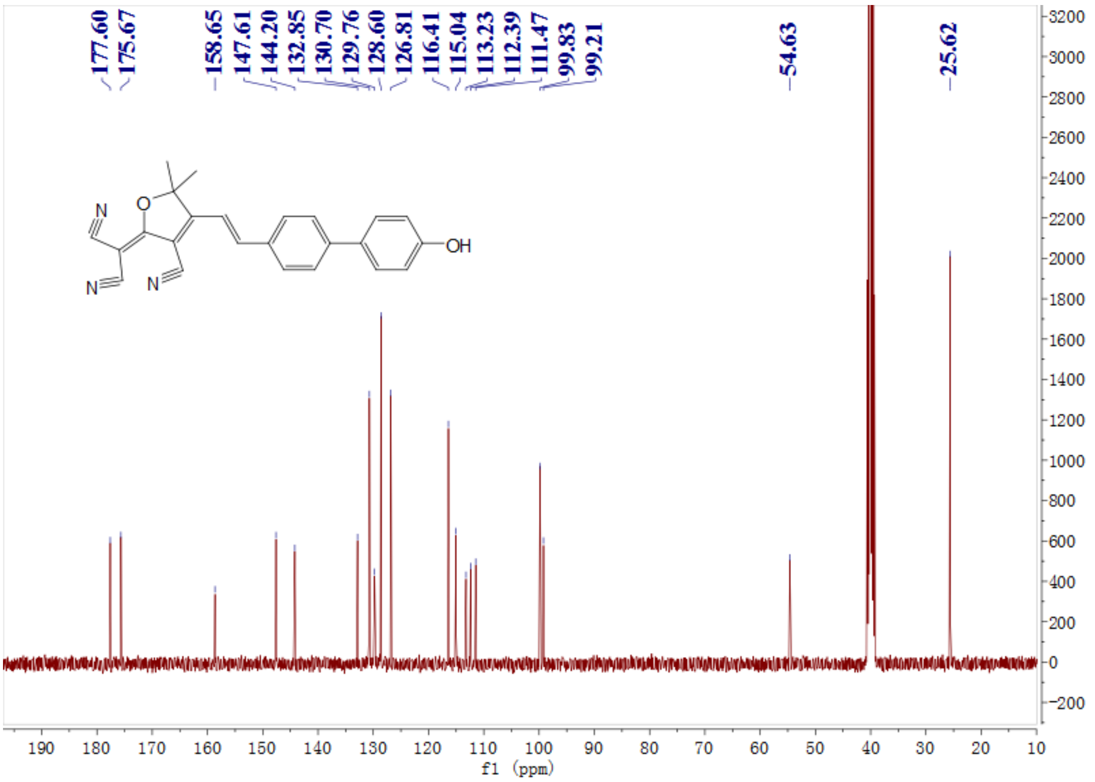

Figure S12. ^{13}C NMR (101 MHz, DMSO-d_6) spectrum of TCF-VIS1.

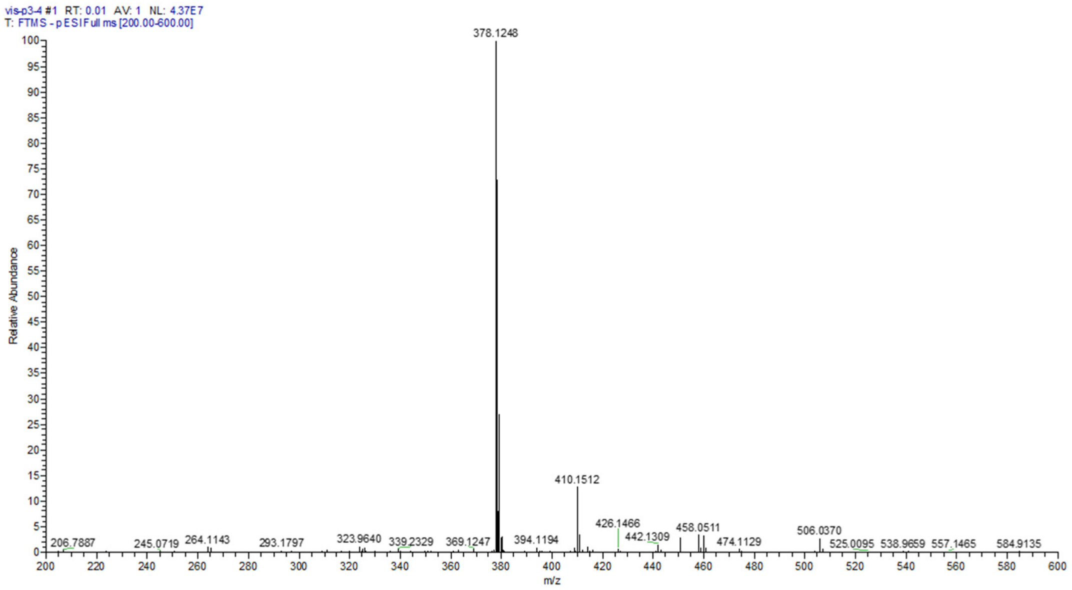

Figure S13. HRMS analysis of the reaction solution of TCF-VIS1.

Table S2. Previous viscosity probes and this work

Probes	λ_{ex}/nm	λ_{em}/nm	Stokes shift/nm	Sensitivity	Response multiple	Bioimaging application	Ref.
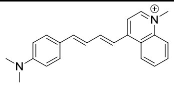	560	670	110	0.958	167-folds	Cell, Zebrafih，Liver tissues	1
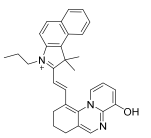	617	650	33	0.71	16.2-folds	Cell	2
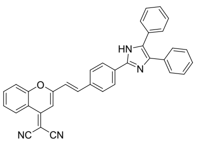	461	624	163	0.419	7.5-folds	Cell and mice	3
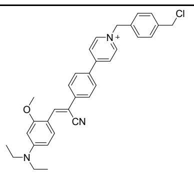	470	650	180	0.678	92-folds	Cell	4
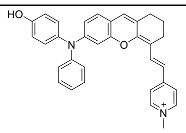	582	720	138	0.725	157-folds	Cell and mice	5
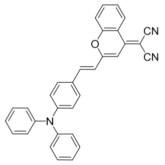	520	696	176	0.549	30-folds	Blood vessel, Mice	6
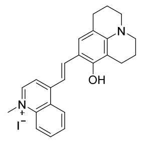	610	734	124	0.933	175-folds	Cells	7
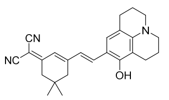	510	675	165	2.3	400-folds	Cells, Zebra fishes	8
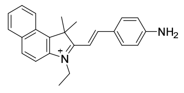	525	583	58	0.58	66-folds	Cell	9
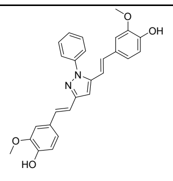	350	400	50	0.97	28.6-folds	Cells, Zebra fishes	10
This work	460	644	184	0.924	78-folds	Cell and mice	

References

1. Zhang Y, Li Z, Hu W, Liu Z. A mitochondrial-targeting near-infrared fluorescent probe for visualizing and monitoring viscosity in live cells and tissues. *Anal Chem.* **2019**, 91, 10302-10309.
2. Wei YF, Weng XF, Sha XL, Sun R, Xu YJ, Ge JF. Simultaneous imaging of lysosomal and mitochondrial viscosity under different conditions using a NIR probe. *Sensor. Actuat. B. Chem.* **2021**, 326, 128954.
3. Liu Y, Ma Y, Gao W, Ma S, Lin W. Construction of a fluorescent probe with large stokes shift and deep red emission for sensing of the viscosity in hyperglycemic mice. *Dyes Pigments.* **2021,** *195*, 109674.
4. Wang X, Fan L, Wang S, Zhang Y, Li F, Zan Q, Lu W, Shuang S, Dong C. Real-time monitoring mitochondrial viscosity during mitophagy using a mitochondria-immobilized near-infrared aggregation-induced emission probe. *Anal Chem.* **2021**, 93, 3241-3249.
5. Fan L, Zan Q, Wang X, Yu X, Wang S, Zhang Y, Yang Q, Lu W, Shuang S, Dong C. A mitochondria-targeted and viscosity-sensitive near-infrared fluorescent probe for visualization of fatty liver, inflammation and photodynamic cancer therapy. *Chem. Eng. J.* **2022**, 449, 137762.
6. Yin J, Kong X, Lin W. Noninvasive cancer diagnosis *in vivo* based on a viscosity-activated near-infrared fluorescent probe. *Anal Chem*. **2021**, 93, 2072-2081.
7. Zhang S, Zhang Y, Zhao L, Xu L, Han H, Huang Y, Fei Q, Sun Y, Ma P, Song D. A novel water-soluble near-infrared fluorescent probe for monitoring mitochondrial viscosity. *Talanta.* **2021**, 233, 122592.
8. Fu M, Shen W, Chen Y, Yi W, Cai C, Zhu L, Zhu Q. A highly sensitive red-emitting probe for the detection of viscosity changes in living cells, zebrafish, and human blood samples. *J. Mater. Chem. B.* **2020**, 8, 1310-1315.
9. Chen B, Li C, Zhang J, Kan J, Jiang T, Zhou J, Ma H. Sensing and imaging of mitochondrial viscosity in living cells using a red fluorescent probe with a long lifetime. *Chem. Commun.* **2019**, 55, 7410-7413.
10. Du W, Gu Y, Zhou X, Wang Z, Wang S. Rational design and comparison of three curcumin-based fluorescent probes for viscosity detection in living cells and zebrafish. *Analyst*. **2024**, 149, 789-799.
